# Supplementary figures and images for: TRIM26 alleviates fatal immunopathology by regulating inflammatory neutrophil infiltration during Candida infection
Source: PLoS Pathog. 2024 Jan 2;20(1):e1011902. doi: 10.1371/journal.ppat.1011902 (PMC10786383; doi:10.1371/journal.ppat.1011902)

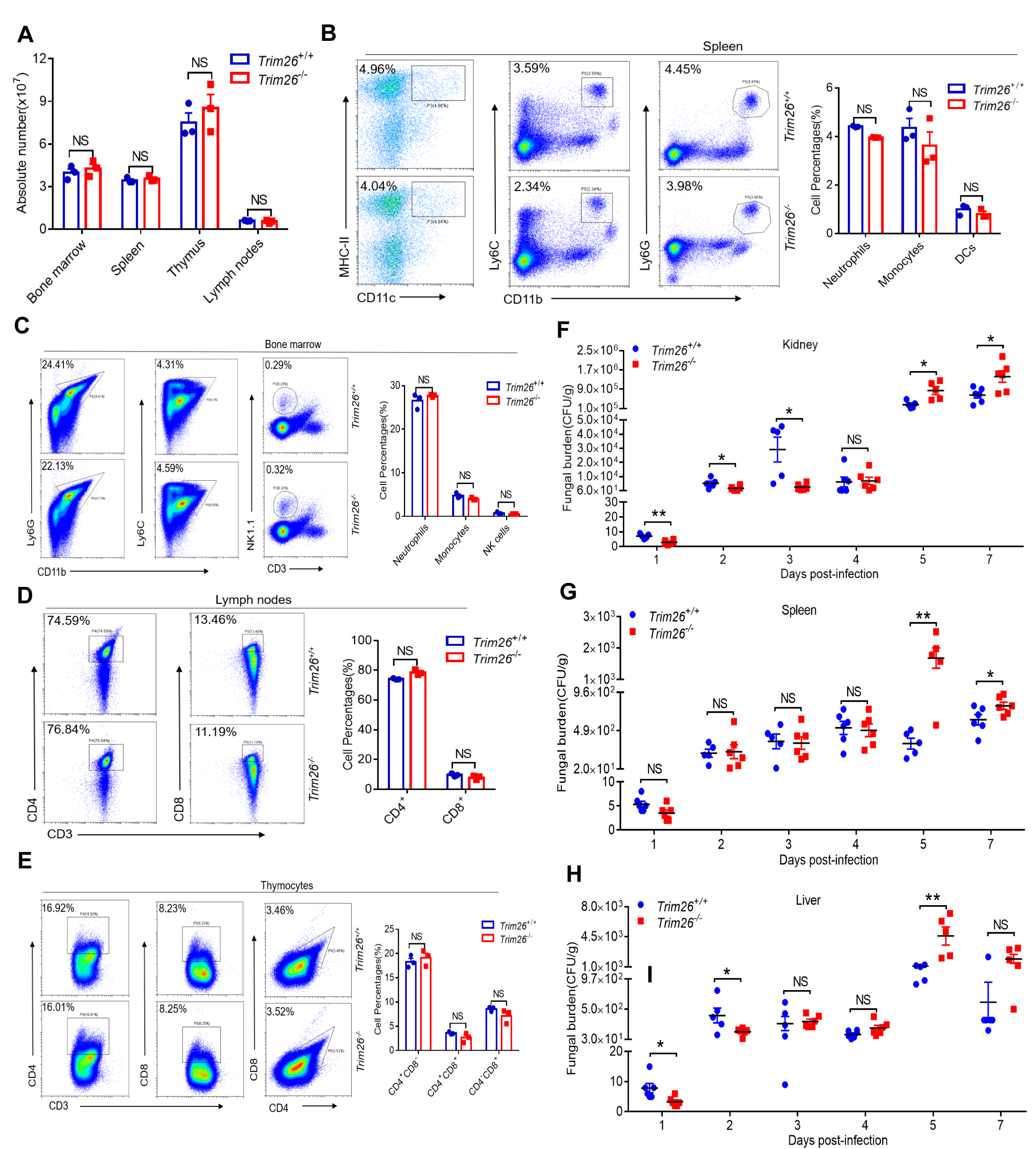

Supplement: S1 Fig — (A)The absolute cell number of immune organs were detected. (B-E) Cells were isolated from spleen (B), bone marrows (C), lymph nodes (D), and thymocytes (E) of WT mice or Trim26-KO mice, followed by flow cytometry analysis for the indicated immune cell populations. (F-H) Wild-type control mice or Trim26-deficient mice were intravenous injected with live C. albicans (2×10^5^ cfu/100 μL 1×PBS), the fungal burden in the spleen, kidney and liver after 1, 2, 3, 4, 5 and 7 days post-infection were shown.*: P<0.05; **: P<0.01; ***: P<0.001 based on unpaired two-tailed t test (A-E and F-H). NS: no significance. Data are shown as mean ± SD. Each point represents a single mouse (A-E and F-H).In A, F-H, one representative experiment of three independent experiments is shown. In B-E, one representative experiment of two independent experiments is shown. (TIF) [file ppat.1011902.s002.tif]

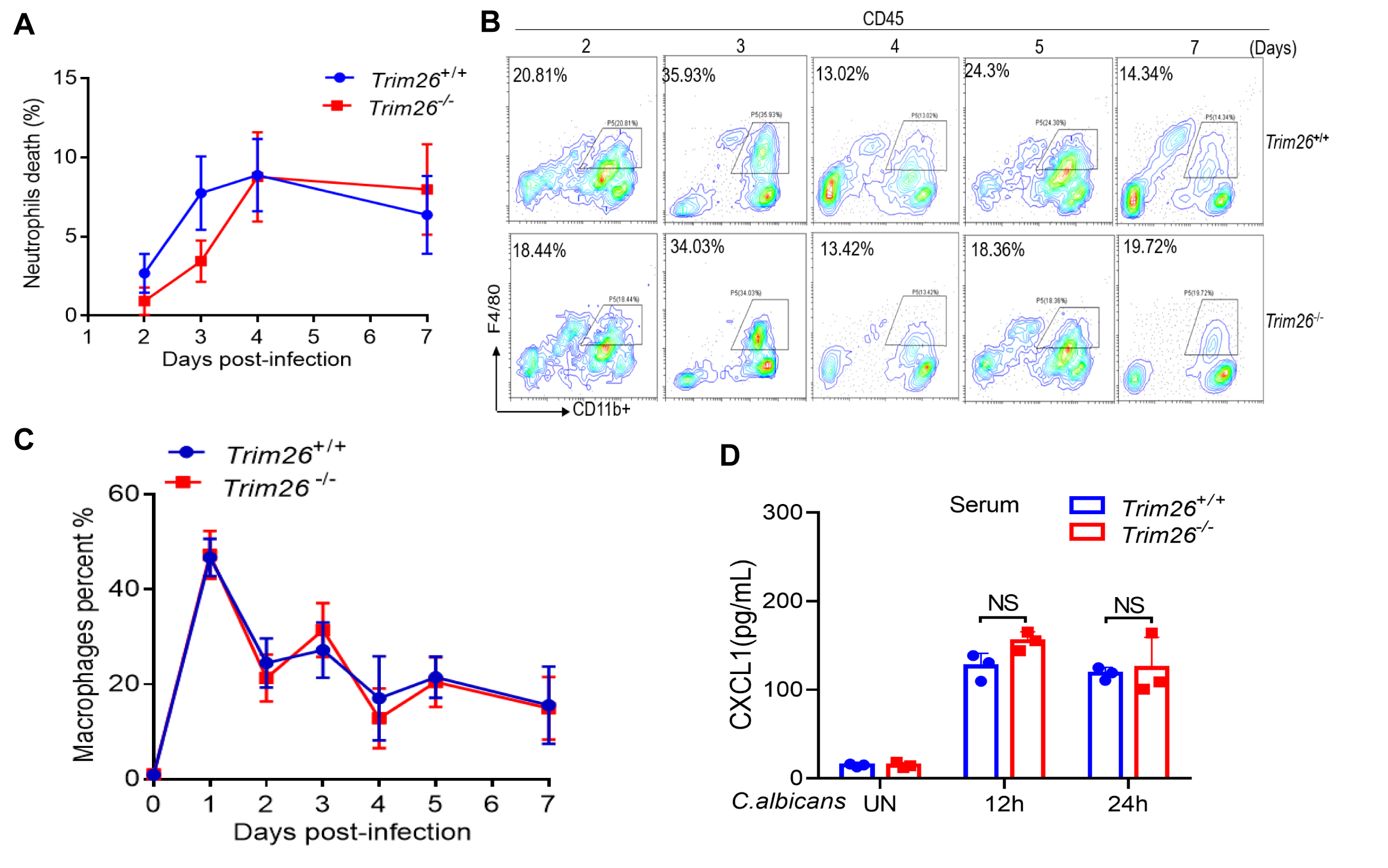

Supplement: S2 Fig — (A-D) WT mice or Trim26 KO mice were intravenously injected with live C. albicans (1×10^5^ cfu/100μL 1×PBS), and mice were euthanized at the indicated day after infection. Cells from kidneys were stained for flow cytometry analysis. (A) The death rate of neutrophils from kidneys were analysed by flow cytometry. (B) Representative contour plot for CD45+F4/80+CD11b+ macrophages and their percentages were presented (C). (D) The level of CXCL1 concentration in serum was determined by ELISA. *: P<0.05; **: P<0.01; ***: P<0.001 based on two-tailed unpaired t-test. NS: no significance. Data are shown as mean ± SD. In A-C, one representative experiment of three independent experiments is shown. Each point represents a single mouse (D). In D, one representative experiment of two independent experiments is shown. (TIF) [file ppat.1011902.s003.tif]

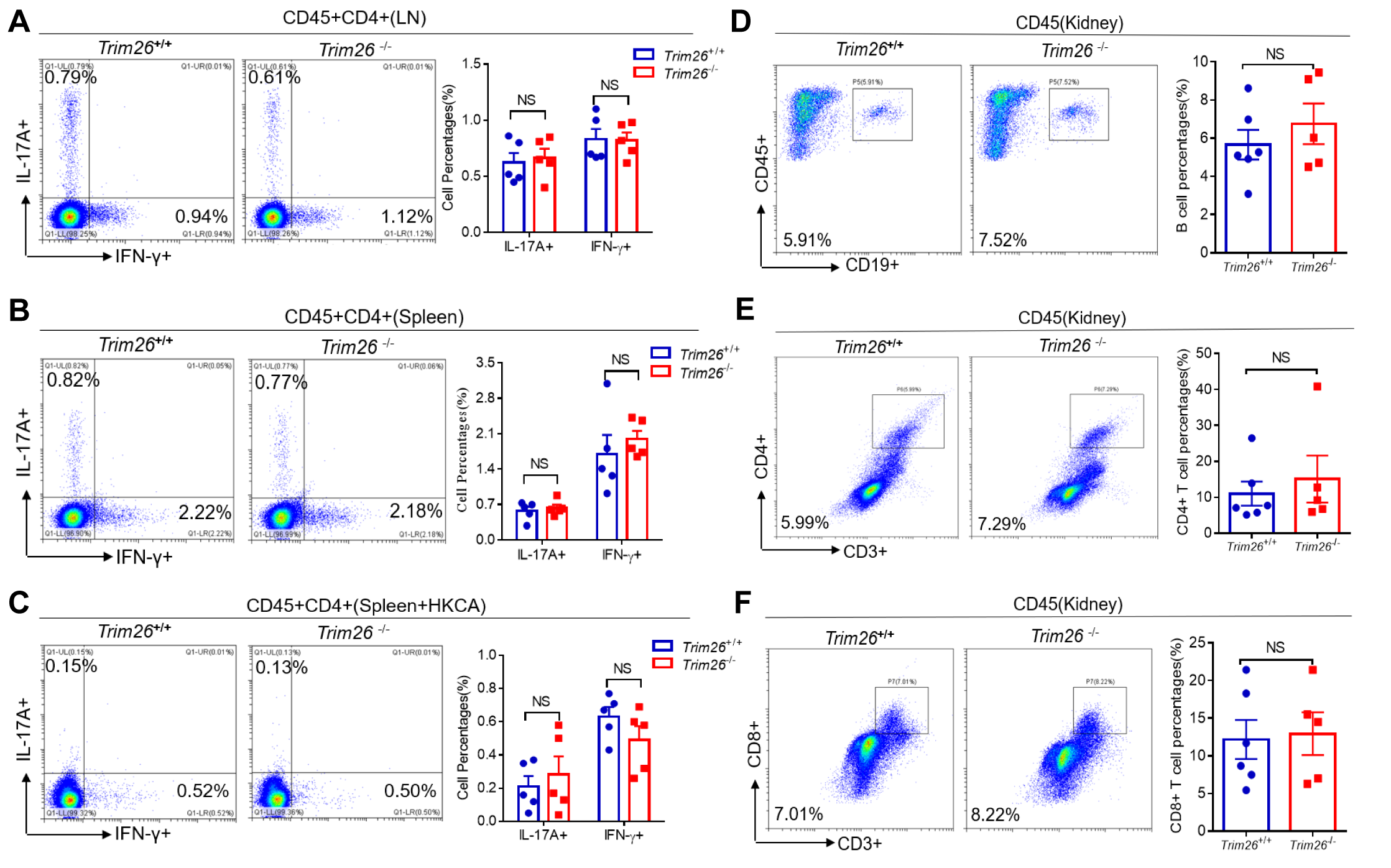

Supplement: S3 Fig — (A-C) WT mice or Trim26 KO mice were intravenously injected with live C. albicans (1×10^5^ cfu/100μL 1×PBS), and mice were euthanized at day 7 after infection. Th1 and Th17 cells from lymph nodes (A) and spleen (B) were stained as indicated for flow cytometry analysis. (C) Splenic cells isolated from the WT mice or Trim26 KO mice infected with C. albicans (1×10^5^ cfu/100 μL 1×PBS) for 5 days, and stimulated with HKCA-Y (MOI = 10) for 2 days. Intracellular staining of Th1 (IFN-γ) and Th17 (IL-17A) were determined with flow cytometry. (D-F) WT mice or Trim26 KO mice were intravenously injected with live C. albicans (1×10^5^ cfu/100 μL 1×PBS), and mice were euthanized at day 7 after infection. B cells (D), CD4+ T cells (E) and CD8+ T cells (F) from kidney were stained as indicated for flow cytometry analysis. *: P<0.05; **: P<0.01; ***: P<0.001 based on two-tailed unpaired t-test. NS: no significance. Data are shown as mean ± SD. In A-F, each point represents a single mouse. In A-F, one representative experiment of three independent experiments is shown. (TIF) [file ppat.1011902.s004.tif]

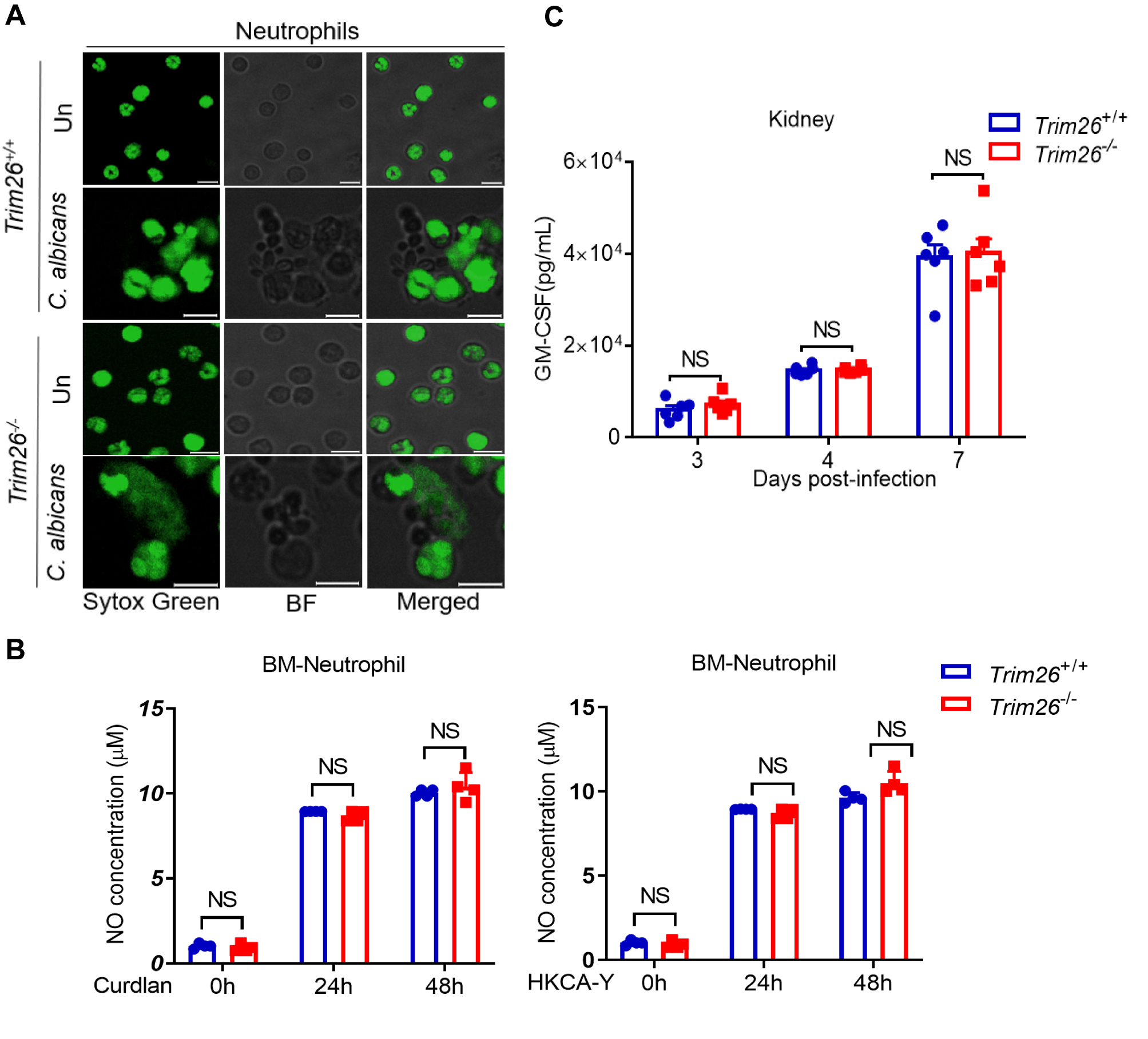

Supplement: S4 Fig — (A) WT mice or Trim26-KO BM neutrophils cells were stimulated heat-killed C. albicans (MOI = 10), then the neutrophil extracellular traps of neutrophils was showed with staining Sytox Green. Scale bars, 10 μm. (C) WT mice or Trim26 KO mice were intravenously injected with live C. albicans (1×10^5^ cfu/100 μL 1×PBS), and mice were euthanized at the indicated day after infection. GM-CSF concentration in kidney was measured by ELISA analysis. (B) WT mice or Trim26-KO BM neutrophils cells were stimulated curdlan (100 μg/mL) or heat-killed C. albicans (MOI = 10) for the indicated time, followed by measurement of NO production via a Nitric Oxide Assay Kit. *: P<0.05; **: P<0.01; ***: P<0.001 based on two-tailed unpaired t-test. NS: no significance. Data are shown as mean ± SD. Each point represents a single mouse (B-C). In A, one representative experiment of two independent experiments is shown. In B-C, one representative experiment of three independent experiments is shown. (TIF) [file ppat.1011902.s005.tif]

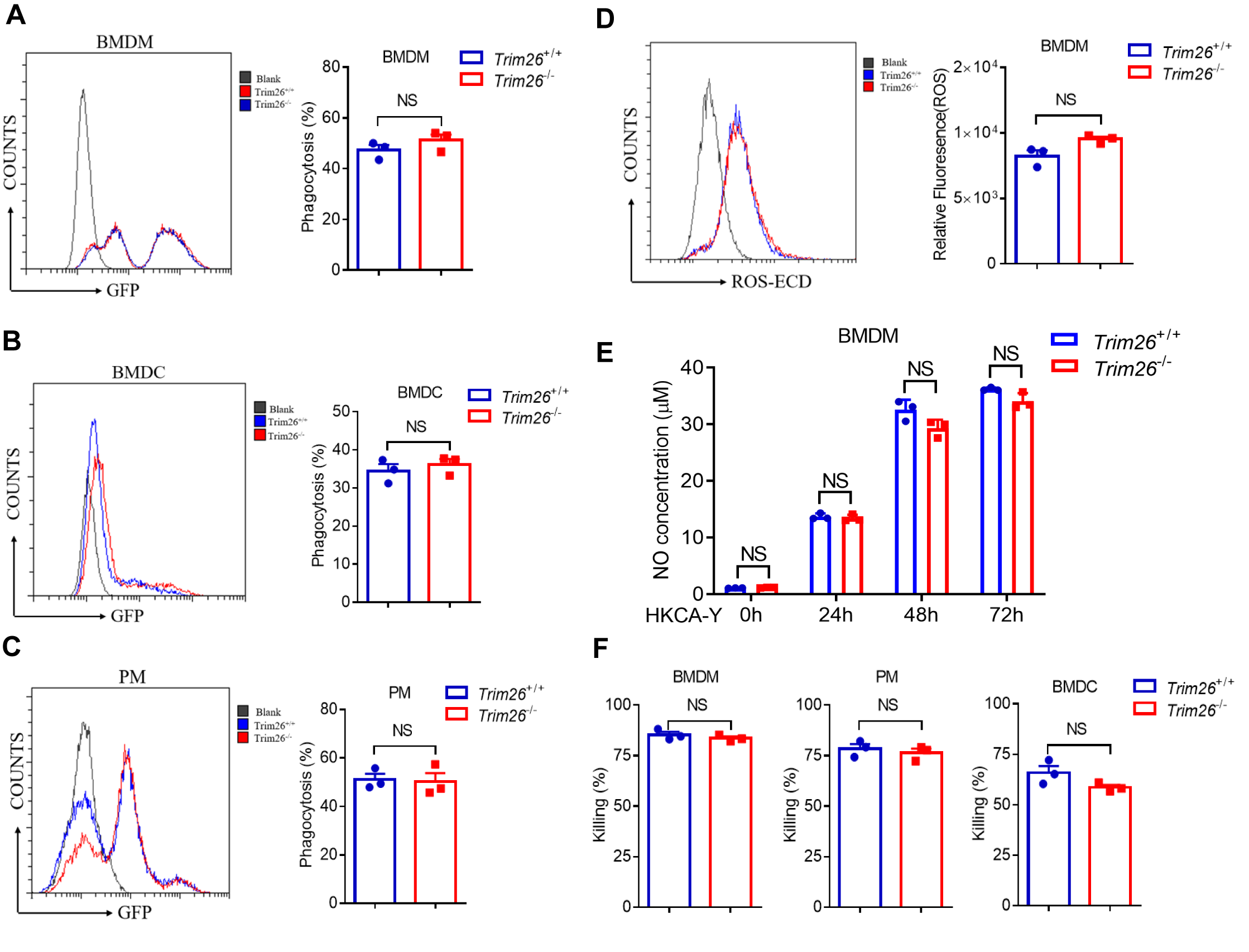

Supplement: S5 Fig — (A-C) Phagocytosis of WT mice or Trim26-KO mice BMDMs (A), BMDCs (B) or PMs (C) was evaluated by the method described in Materials and Methods. (D) WT mice or Trim26-KO BMDMs cells were stimulated heat-killed C. albicans (MOI = 10) for 30 min, followed by measurement of ROS production via a fluorescent ROS probe. (E) WT mice or Trim26-KO BMDMs were stimulated with heat-inactivated C. albicans (MOI = 10) for the indicated time. NO production in culture supernatants at the indicated time point was measured by nitrite assay kit. (F) Killing capacity of WT mice or Trim26-KO BMDMs, PM and BMDCs as assessed by co-culture with C. albicans. Assays were performed in triplicate. *: P<0.05; **: P<0.01; ***: P<0.001 based on two-tailed unpaired t-test. NS: no significance. Data are shown as mean ± SD. In A-F, one representative experiment of three independent experiments is shown. (TIF) [file ppat.1011902.s006.tif]
