# Supplementary material for: TRIM26 alleviates fatal immunopathology by regulating inflammatory neutrophil infiltration during Candida infection
Source: PLoS Pathog. 2024 Jan 2;20(1):e1011902. doi: 10.1371/journal.ppat.1011902 (PMC10786383; doi:10.1371/journal.ppat.1011902)
Supplement: S1 Table — (DOCX) [file ppat.1011902.s007.docx]

**S1 Table. qPCR primers used in this study**

**qPCR primers**

| **Name** | **Purpose** | **Sequence(5'-3')** | **Source** |
| --- | --- | --- | --- |
| *β-actin* FP | For qPCR of *β-actin* | CCACACCCGCCACCAGTTCG | [1] |
| *β-actin* RP | For qPCR of *β-actin* | TACAGCCCGGGGAGCATCGT | [1] |
| *IL-6* FP | For qPCR of *IL-6* | ACAACCACGGCCTTCCCTAC | [2] |
| *IL-6* RP | For qPCR of *IL-6* | CATTTCCACGATTTCCCAGA | [2] |
| *IL-1β* FP | For qPCR of *IL-1β* | ACCTTCCAGGATGAGGACATGA | [2] |
| *IL-1β* RP | For qPCR of *IL-1β* | AACGTCACACACCAGCAGGTTA | [2] |
| *TNF-α* FP | For qPCR of *TNF-α* | GCCACCACGCTCTTCTGTCT | [2] |
| *TNF-α* RP | For qPCR of *TNF-α* | TGAGGGTCTGGGCCATAGAAC | [2] |
| *IL-10* FP | For qPCR of *IL-10* | TGGGTGAGAAGCTGAAGACCCT | this study |
| *IL-10* RP | For qPCR of *IL-10* | CCTGCTCCACTGCCTTGCTC | this study |
| *Ngal* FP | For qPCR of *Ngal* | GATGCGCAGAGACCCAATG | this study |
| *Ngal* RP | For qPCR of *Ngal* | TCTGGCAACAGGAAAGATGGA | this study |
| *Kim1* FP | For qPCR of *Kim1* | ACATATCGTGGAATCACAACGAC | [3] |
| *Kim1* RP | For qPCR of *Kim1* | ACAAGCAGAAGATGGGCATTG | [3] |
| *Icam1* FP | For qPCR of *Icam1* | CCCCGCAGGTCCAATTC | this study |
| *Icam1*RP | For qPCR of *Icam1* | CAGAGCGGCAGAGCAAAAG | this study |
| *P-Selectin* FP | For qPCR of *P-Selectin* | GGGCTCCTCCCGAATGTC | this study |
| *P-Selectin* RP | For qPCR of *P-Selectin* | GCTGAACGCAGGTCATGGA | this study |
| *Ly6g* FP | For qPCR of *Ly6g* | CTTCCCATCTGCCCCACTAC | this study |
| *Ly6g* RP | For qPCR of *Ly6g* | CTTCCCATCTGCCCCACTAC | this study |
| *S100a8* FP | For qPCR of *S100a8* | GGAAATCACCATGCCCTCTAC | this study |
| *S100a8* RP | For qPCR of *S100a8* | CCTTGTGGCTGTCTTTGTGAG | this study |
| *Mpo* FP | For qPCR of *Mpo* | GAGAGTCGTGTTGGAAGGTGG | this study |
| *Mpo* RP | For qPCR of *Mpo* | TGCTCAAATAGTCGCTCCCG | this study |
| *CXCL-1* FP | For qPCR of *CXCL-1* | TGGCTGGGATTCACCTCAAGAACA | this study |
| *CXCL-1* RP | For qPCR of *CXCL-1* | TGTGGCTATGACTTCGGTTTGGGT | this study |
| *CXCL-2* FP | For qPCR of *CXCL-2* | GTGAACTGCGCTGTCAATGC | this study |
| *CXCL-2* RP | For qPCR of *CXCL-2* | GCTTCAGGGTCAAGGCAAAC | this study |
| *IL-2* FP | For qPCR of *IL-2* | CTGGAGCAGCTGTTGATGGA | this study |
| *IL-2* RP | For qPCR of *IL-2* | TCAAATCCAGAACATGCCGC | this study |
| *Ccl2* FP | For qPCR of *Ccl2* | TTGGGATCATCTTGCTGGTG | this study |
| *Ccl2* RP | For qPCR of *Ccl2* | TCTGGGCCTGCTGTTCACA | this study |
| *IL-12*a FP | For qPCR of *IL-12*a | AAGCTCTGCATCCTGCTTCAC | this study |
| *IL-12*a RP | For qPCR of *IL-12*a | GATAGCCCATCACCCTGTTGA | this study |
| *Cat* FP | For qPCR of *Cat* | GCTGAGAAGCCTAAGAACGCAAT | this study |
| *Cat* RP | For qPCR of *Cat* | CCCTTCGCAGCCATGTG | this study |
| *Sod2* FP | For qPCR of *Sod2* | CCTGCTCTAATCAGGACCCATT | this study |
| *Sod2* RP | For qPCR of *Sod2* | CGTGCTCCCACACGTCAAT | this study |
| *Nox4* FP | For qPCR of *Nox4* | AGCATCTGCATCTGTCCTGAAC | this study |
| *Nox4* RP | For qPCR of *Nox4* | CCTGCTAGGGACCTTCTGTGA | this study |
| *Mmp8* FP | For qPCR of *Mmp8* | CACACTCCGTGGGGAGATTT | this study |
| *Mmp8* RP | For qPCR of *Mmp8* | CCTGAAGACCGTTGGGTAGG | this study |

FP = forward primer

RP = reverse primer

References:

[1].Wang X, Zhang H, Shao Z, Zhuang W, Sui C, Liu F, et al. TRIM31 facilitates K27-linked polyubiquitination of SYK to regulate antifungal immunity. Signal Transduct Target Ther. 2021 Aug 6;6(1):298. PubMed PMID: 34362877; PubMed Central PMCID: PMC8342987.

[2].Chen X, Zhang H, Wang X, Shao Z, Li Y, Zhao G, et al. OTUD1 Regulates Antifungal Innate Immunity through Deubiquitination of CARD9. J Immunol. 2021 Apr 15;206(8):1832-1843. PubMed PMID: 33789983.

[3].Majer O, Bourgeois C, Zwolanek F, Lassnig C, Kerjaschki D, Mack M, et al. Type I interferons promote fatal immunopathology by regulating inflammatory monocytes and neutrophils during Candida infections. PLoS Pathog. 2012;8(7):e1002811. PubMed PMID: 22911155; PubMed Central PMCID: PMC3406095.
