# Supplementary material for: TRIM26 alleviates fatal immunopathology by regulating inflammatory neutrophil infiltration during Candida infection
Source: PLoS Pathog. 2024 Jan 2;20(1):e1011902. doi: 10.1371/journal.ppat.1011902 (PMC10786383; doi:10.1371/journal.ppat.1011902)
Supplement: Attachment — Submitted filename: 4 point-by-point response.docx [file ppat.1011902.s010.docx]

Point-by-point response

We are thankful for both editor and reviewers’ comments and suggestions, which helped us to further improve the quality of this manuscript. Please find our point-by-point response below:

Editor Comments:

Thank you for submitting your paper, "TRIM26 alleviates fatal immunopathology by regulating inflammatory neutrophils infiltration during Candida infections", to PLOS Pathogens. We have checked over your submission and, before we can assign your manuscript to the Editor, we ask that you first please make the following modifications:

1) Please ensure that the CRediT author contributions listed for every co-author are completed accurately and in full. At this stage, "All Authors" require contributions. Please ensure that the full contributions of each author are acknowledged in the ""Add/Edit/Remove Authors"" section of our submission form.

The list of CRediT author contributions may be found here:

https://journals.plos.org/plospathogens/s/authorship#loc-author-contributions

We are thankful for the editor’s comment and suggestion, and we revised and refined the author contributions section to the manuscript of the article.

2) Please amend your detailed Financial Disclosure statement. This is published with the article, therefore should be completed in full sentences and contain the exact wording you wish to be published.

i). State the initials, alongside each funding source, of each author to receive each grant.

ii). State what role the funders took in the study. If the funders had no role in your study, please state: “The funders had no role in study design, data collection and analysis, decision to publish, or preparation of the manuscript.”

iii). If any authors received a salary from any of your funders, please state which authors and which funders.

We are thankful for the editor’s suggestion, and we amended the funding section to the manuscript of the article. The funders had no role in study design, data collection and analysis, decision to publish, or preparation of the manuscript.

3) Please ensure that the funders and grant numbers match between the Financial Disclosure field and the Funding Information tab in your submission form. Note that the funders must be provided in the same order in both places as well.Currently, the grant numbers " 32230033, ZR201911140289" are missing from the Funding Information.

We are thankful for the editor’s suggestion, and we have confirmed the correctness of the funders and grant numbers. We have confirmed that the funders and grant numbers match between the Financial Disclosure field and the Funding Information tab in ours submission form.

4) We do not publish any copyright or trademark symbols that usually accompany proprietary names, eg (R), (C), or TM (e.g. next to drug or reagent names). Please remove all instances of trademark/copyright symbols throughout the text, including SuperScript® on line no 701

We are thankful for the editor’s suggestion, and we removed all instances of trademark/copyright symbols throughout the text.

5) Please insert an Ethics Statement at the beginning of your Methods section, under a subheading 'Ethics Statement'. It must include:

1) The name(s) of the Institutional Review Board(s) or Ethics Committee(s)

2) The approval number(s), or a statement that approval was granted by the named board(s)

We are thankful for the editor’s suggestion, and we inserted an Ethics Statement at the beginning of the Methods section.

6) Some material included in your submission may be copyrighted. According to PLOS’s copyright policy, authors who use figures or other material (e.g., graphics, clipart, maps) from another author or copyright holder must demonstrate or obtain permission to publish this material under the Creative Commons Attribution 4.0 International (CC BY 4.0) License used by PLOS journals. Please closely review the details of PLOS’s copyright requirements here: PLOS Licenses and Copyright. If you need to request permissions from a copyright holder, you may use PLOS's Copyright Content Permission form.

Please respond directly to this email and provide any known details concerning your material's license terms and permissions required for reuse, even if you have not yet obtained copyright permissions or are unsure of your material's copyright compatibility. Once you have responded and addressed all other outstanding technical requirements, you may resubmit your manuscript within Editorial Manager.

Potential Copyright Issues:

1) [Figure 2]: Please confirm (a) that you are the photographer; or (b) provide written permission from the photographer to publish the photo(s) under our CC BY 4.0 license.

2) [Figure4,6]: Please confirm whether you drew the images / clip-art within the figure panels by hand. If you did not draw the images, please provide (a) a link to the source of the images or icons and their license / terms of use; or (b) written permission from the copyright holder to publish the images or icons under our CC BY 4.0 license. Alternatively, you may replace the images with open source alternatives. See these open source resources you may use to replace images / clip-art:

- https://commons.wikimedia.org

- https://openclipart.org/

We are thankful for the editor’s comment and suggestion. We confirmed the image of Figure 2D was taken by the first author himself. We have replaced the images in Figure 4A and Figure 6H with open source resources, all of which are from <https://commons.wikimedia.org> and https://openclipart.org/.

Editor Comments:

Your manuscript "TRIM26 alleviates fatal immunopathology by regulating inflammatory neutrophils infiltration during *Candida infections*" for consideration at PLOS Pathogens. As with all papers reviewed by the journal, your manuscript was reviewed by members of the editorial board and by several independent reviewers. In light of the reviews (below this email), we would like to invite the resubmission of a significantly-revised version that takes into account the reviewers' comments.

The manuscript has been reviewed by three experts in the field, and the critiques are mixed. There are several concerns that need to be addressed.

We are thankful for the editor’s comments, and we performed the experiments suggested by the reviewers to address the reviewers’ concerns.

1. As reviewer 1 has pointed out, many of the changes in chemokines and cytokines may be caused by differences in fungal burden. Therefore, it is imperative to revisit these experiments and analyze the soluble mediators when fungal burdens are similar if not identical.

We are thankful for the editor’s summarization, and we revisited the changes in chemokines and cytokines when fungal burden was similar in the revised manuscript (Figure 2F, Figure 3F-G, Supplementary Figure 1G-I).

2. To validate the role of CXCL1, the authors should neutralize it or block it and assess the impact on fungal burden. That piece of data will confirm that CXCL1 is the cause of the changes in neutrophil attraction.

We are thankful for the editor’s suggestion, and we validated the role of CXCL1 in *Trim26^–/–^* mice by using CXCR1 and CXCR2 inhibitor Reparixin to block the process of CXCL1 recruiting neutrophils, and please find the data in the Figure 3I-L of the revised manuscript.

3. The authors need to determine how TRIM 26 regulates CXCL1.

We are thankful for the editor’s suggestion, and we determined the mechanism how Trim26 regulated the expression of CXCL1, and please find the data in the Figure 6A-G of the revised manuscript.

4. The authors need to reconsider their hypothesis that immunopathology is the cause of the more severe disease in the kidneys. The increase in fungal burden may be a cue for exaggerated neutrophil influx. The authors need to discriminate between the effect of the burden versus heightened recruitment. Again, examining the neutrophil response at a time when the burdens are similar would be needed. Or, infecting mice with different numbers of fungi to equalize the burden and then examine the neutrophil response.

We are thankful for the editor’s suggestion, and we examined the neutrophils response at a time when the fungal burdens were similar, and please find the data in the Figure 3H and Supplementary Figure 1G-I of the revised manuscript.

5. Perform additional experiments to identify the root cause of the killing defect by neutrophils. As mentioned by reviewer 3 and I agree, the differences in ROS are not striking. And an n=2 is underwhelming.

We are thankful for the editor’s suggestion, and we performed additional experiments to explore the cause of the deficiency of neutrophils killing, containing phagocytosis, neutrophil extracellular traps (NETs), ROS and NO production. We only found the ROS production was significantly reduced in *Trim26^–/–^* neutrophils compared to *Trim26^+/+^* neutrophils. ROS plays an essential role in regulating various physiological functions of living organisms. The intracellular content of ROS is relatively redox homeostasis. Mitochondrial electron transport chain (Mito-ETC), ER system, and NOX complex are the major reaction sites for intracellular ROS generation. To prevent the harmful effects of excessive ROS, various ROS-scavenging enzymes are also available in cells to participate in the downregulation of oxidative stress, thus keeping ROS content within normal ranges. ROS production in response to fungal stimulation was not striking but had significantly alteration in other papers (PMC6209141, PMC4975523 and PMID: 35022276). We also increased the number of mice samples and presented pooled data for the ROS production, and please find the data in the Figure 4I-K, Figure S3D, and S3E of the revised manuscript.

6. Instead of bone marrow macrophages, the authors need to examine CXCL1 by renal macrophages. That is key to understanding the organ-specific effect.

We are thankful for the editor’s suggestion, and we examined the renal macrophages data of CXCL1 response stimulated by curdlan (Figure 5E in the revised manuscript).
Reviewer's Responses to Questions

**Part I - Summary**
Please use this section to discuss strengths/weaknesses of study, novelty/significance, general execution and scholarship.

Reviewer #1: The manuscript by Zhao and colleagues analyzed the function of Trim26 during systemic candidiasis. The authors found that Trim26-deficient mice are more susceptible to lethal infection. The authors show that Trim26 restricts immunopathology by restricting CXCL1 secretion in macrophages and DCs (in vitro) to prevent excessive neutrophil influx. Some additional experiments are required to support the author’s findings.

Reviewer #2: This manuscript examines the role of Trim26 in pathogenesis in a murine model of invasive candidiasis. The finding that Trim26 deficient animals have higher proinflammatory cytokines and neutrophil recruitment in the kidneys is interesting. This is further linked to CXCL1 and CXCL2 production. Bone marrow transplant studies add rigor. Appropriate controls are included.

Reviewer #3: In this paper, the authors examine the role of Trim26 in host defense against systemic candidiasis. the authors show that Trim26-deficient mice have increased mortality and fungal burden after infection associated with increased renal failure. the susceptibility maps to the hematopoietic compartment. the authors show increase neutrophil accumulation in the knockout kidneys associated with increase neutrophil chemokines and a small change in killing capacity in knockout neutrophils. Upon exposure of bone marrow mononuclear phagocytes to curdlan Trim26 KO cells produced more chemokines. Although the work is of interest, there are several concerns with the conclusions of the manuscript and the presented data as outlined below:

**Part II – Major Issues: Key Experiments Required for Acceptance**
Please use this section to detail the key new experiments or modifications of existing experiments that should be absolutely required to validate study conclusions.

Generally, there should be no more than 3 such required experiments or major modifications for a "Major Revision" recommendation. If more than 3 experiments are necessary to validate the study conclusions, then you are encouraged to recommend "Reject".

Reviewer #1: -The authors provide expression data from several cytokines. However, increased fungal burden (Fig. 1C) results in many cases to increased inflammation. The time point when fungal burden was measured is not indicated in the figure legend. In this context, is CXCL1 expression increased in Trim26 KO mice early during infection (12/24 hours)?
We are thankful for the reviewer’s suggestion, and we added the time point when fungal burden was measured in the figure legend. We examined the CXCL1 expression level in Trim26 KO mice during early infection (12/24 hours), and please find the data in the Figure S3A of the revised manuscript.

-Based on in vivo and in vitro data the authors speculate that CXCL1 is the driver for increased neutrophil influx and immunopathology in the Trim26 KO mice. To verify this, the authors should deplete CXCL1 during infection and verify the phenotypes.
We are thankful for the reviewer’s suggestion, and we validated the role of CXCL1 in Trim26–/– mice by using CXCR1 and CXCR2 inhibitor Reparixin to block the process of CXCL1 recruiting neutrophils, and please find the data in the Figure 3I-L of the revised manuscript.

-The authors show that Trim26 KO BMDMs/DCs increases CXCL1 expression. However, it is unclear how Trim26 negatively regulates/restricts CXCL1 expression. The authors show look into the signaling pathways (CARD9–BCL10–MALT1, NF-κB, and MAPK) to determine involvement.

We are thankful for the reviewer’s suggestion, and we determined the mechanism how Trim26 regulated the expression of CXCL1, and please find the data in the Figure 6A-G of the revised manuscript.

Reviewer #2: The introduction should be updated to include references to original data (instead of reviews). For discussion of known immune responses, please clarify if these are for murine or human studies. Please clarify systemic infection versus mucosal candidiasis.
We are thankful for the reviewer’s suggestion, and we updated the original references in introduction (pages 4, lines 53-54, and lines 60 ), and clarified the murine or human studies and systemic infection versus mucosal candidiasis in the discussion part of the revised manuscript (pages 15-17, lines 379, lines 382-385, lines 401-402, lines 406-407, lines 410-411).

Include biological replicates in figure legends.
We are thankful for the reviewer’s suggestion, and we added biological replicates in figure legends of the revised manuscript. The data represent the means ± SDs of at least 2-3 replicates.

Explain why curdlan was used for studies.

We are thankful for the reviewer’s suggestion. Curdlan is a high molecular weight linear polymer consisting of pure β-(1->3)-linked glucose residues. Curdlan is produced as a water-insoluble polysaccharide by the soil bacterium, *Alcaligenes faecalis*. Curdlan is recognized by the membrane bound dectin-1 receptor leading to the Syk activation and CARD9-dependent activation of NF-κB and MAP kinases1 in BMDCs and BMDMs. Furthermore, Dectin-1 signaling activates the NFAT transcription factor. (PMID: 17450144 and PMID: 19594628)

Reviewer #3: 1-the authors state that the problem in the KO kidneys is excess immunopathology. Actually the data presented here support a model by which Trim26 is important for resistance to infection, not immunopahtology modulation. The KO kidneys have more fungal load and as a result of that there are more neutrophils and more renal damage. The primary problem with KO kidneys and mice is impaired host defense, not immunopathology. Related to this, the sentence in lines 86-88 "In this study, we uncover that Trim26 attenuates fatal immunopathology by restricting inflammatory neutrophils infiltration and limiting the production of proinflammatory cytokines in kidney during Candida infections" is not entirely accurate. Please rephrase.

We are thankful for the reviewer’s suggestion, and we rephrased our statement in the result part of the revised manuscript (pages 5, lines 90, and pages 2, lines 29).

2-related to the above, it is unclear how KO mice cannot defend well against infection. The authors claim that the neutrophils in the KO mice have impaired ROS and killing capacity. However, the ROS MFI difference is at the 10% range, which is quite trivial. The killing defect in renal neutrophils is also small and only based on n of 2 where statistics are performed (which is not appropriate). therefore it remains unclear why the KO mice cannot control the fungal load in the kidney, which would be very important to establish to better understand why Trim26 promotes protective immunity.
We are thankful for the reviewer’s suggestion, and we performed additional experiments to explore the cause of the deficiency of neutrophils killing, containing phagocytosis, neutrophil extracellular traps (NETs), ROS and NO production. We only found the ROS production was significantly reduced in *Trim26^–/–^* neutrophils compared to *Trim26^+/+^* neutrophils. ROS plays an essential role in regulating various physiological functions of living organisms. The intracellular content of ROS is relatively redox homeostasis. Mitochondrial electron transport chain (Mito-ETC), ER system, and NOX complex are the major reaction sites for intracellular ROS generation. To prevent the harmful effects of excessive ROS, various ROS-scavenging enzymes are also available in cells to participate in the downregulation of oxidative stress, thus keeping ROS content within normal ranges. ROS production in response to fungal stimulation was not striking but had significantly alteration in other papers (PMC6209141, PMC4975523 and PMID: 35022276). We also increased the number of mice samples and presented pooled data for the ROS production, and please find the data in the Figure 4H-K, Figure S3D and S3E of the revised manuscript.

3-the authors conclude that increased Cxcl1/Cxcl2 in the KO kidneys is responsible for the greater neutrophils in the kidney. However, the authors do not present any direct evidence for that, rather they present an association of the increased chemokine concentration with increased neutrophils in the kidney. Related to this, the sentences in Lines 197-199 "These data indicate that Trim26 deficiency promotes renal inflammatory neutrophils recruitment via increasing CXCL1 production," is not supported by the data.

We are thankful for the reviewer’s suggestion, and we validated the role of CXCL1 in Trim26^–/–^ mice by using CXCR1 and CXCR2 inhibitor Reparixin to block the process of CXCL1 recruiting neutrophils, and please find the data in the Figure 3I-L of the revised manuscript.

4-the observation that KO mononuclear phagocytes in the bone marrow produce more chemokines is of interest. However, does this also apply to renal macrophages? That would be important to show

We are thankful for the reviewer’s suggestion, and we examined the renal macrophages data of CXCL1 response stimulated by curdlan (Figure 5E in the revised manuscript).

5-in Fig 3A, the authors show % of neutrophils in the kidneys. How about absolute numbers?

We are thankful for the reviewer’s suggestion, and we included absolute numbers of neutrophils in kidney after live *C. albicans* infection at the indicated time in the revised manuscript (Figure 3C).

6- there are many grammatical and typo errors in the paper. Please use the help of a native English speaking person to help ameliorate those errors.

We are thankful for the reviewer’s suggestion, and this manuscript has been polished by native speaker.

**Part III – Minor Issues: Editorial and Data Presentation Modifications**
Please use this section for editorial suggestions as well as relatively minor modifications of existing data that would enhance clarity.

Reviewer #1: (No Response)

Reviewer #2: Grammatical editing is required.

We are thankful for the reviewer’s suggestion, and this manuscript has been polished by native speaker.

Some of the figures are small and difficult to read (example 1D).

We are thankful for the reviewer’s suggestion, and we replaced and amplified the figure 1D in the revised manuscript (Figure 1D)

Line 108: Clarify if the immune cell profile was for uninfected animals.

We are thankful for the reviewer’s suggestion, and we clarified the statement in the revised manuscript (pages 6, lines 115).

The function of Trim26 (E3 ligase) should be mentioned in the abstract.

We are thankful for the reviewer’s suggestion, and we added the function of Trim26 in the abstract in the revised manuscript (pages 2, lines 23-25)

Reviewer #3: (No Response)

Overall, we are really grateful for both of the reviewers and editor’s comments and suggestions for this manuscript, which helped us to improve the quality of this study.
