# Supplementary material for: TRIM26 alleviates fatal immunopathology by regulating inflammatory neutrophil infiltration during Candida infection
Source: PLoS Pathog. 2024 Jan 2;20(1):e1011902. doi: 10.1371/journal.ppat.1011902 (PMC10786383; doi:10.1371/journal.ppat.1011902)
Supplement: Attachment — Submitted filename: 4 point-by-point response.docx [file ppat.1011902.s011.docx]

Point-by-point response

We are thankful for both editor and reviewers’ comments and suggestions, which helped us to further improve the quality of this manuscript. Please find our point-by-point response below:

Editor Comments:

Thank you for submitting your paper, "TRIM26 alleviates fatal immunopathology by regulating inflammatory neutrophil infiltration during Candida infection", to PLOS Pathogens. We have checked over your submission and, before we can assign your manuscript to the Editor, we ask that you first please make the following modifications:

We are thankful for the editor’s comments, and we performed the modifications suggested by the editor’ comments and concerns.

Thank you for editing your submission. Please action the point below so that it can proceed with the review process.

1) Please ensure that the CRediT author contributions listed for every co-author are completed accurately and in full. At this stage, "All Authors" require contributions. Please ensure that the full contributions of each author are acknowledged in the ""Add/Edit/Remove Authors"" section of our submission form.

The list of CRediT author contributions may be found here:

https://journals.plos.org/plospathogens/s/authorship#loc-author-contributions

NOTE:Please update the Authors Contribution in the ONLINE SUBMISSION form.

We are thankful for the editor’s comment and suggestion. It was our negligence. We have updated the Authors Contribution in the ONLINE SUBMISSION form.

2) Some material included in your submission may be copyrighted. According to PLOS’s copyright policy, authors who use figures or other material (e.g., graphics, clipart, maps) from another author or copyright holder must demonstrate or obtain permission to publish this material under the Creative Commons Attribution 4.0 International (CC BY 4.0) License used by PLOS journals. Please closely review the details of PLOS’s copyright requirements here: PLOS Licenses and Copyright. If you need to request permissions from a copyright holder, you may use PLOS's Copyright Content Permission form.

Please respond directly to this email and provide any known details concerning your material's license terms and permissions required for reuse, even if you have not yet obtained copyright permissions or are unsure of your material's copyright compatibility. Once you have responded and addressed all other outstanding technical requirements, you may resubmit your manuscript within Editorial Manager.

Potential Copyright Issues:

Thank you for providing the following information

Kidney icon, https://commons.wikimedia.org/wiki/File:Kidney_icon.svg ( Creative Commons Attribution-Share Alike 3.0 Unported license).

https://commons.wikimedia.org/wiki/File:Equipment_-_Syringe_(5279)_--_Smart-Servier.png

<https://commons.wikimedia.org/wiki/File:C_albicans_budding2.jpg>

<https://commons.wikimedia.org/wiki/File:Macrophage.png>

Neutrophil, <https://commons.wikimedia.org/wiki/File:Neutrophil_(sketch).png>

Kidney icon, <https://commons.wikimedia.org/wiki/File:Kidney_icon.svg>

Kidney no blood, <https://commons.wikimedia.org/wiki/File:Kidney_noblood.png>

Unfortunately, CC BY-SA, CC BY-SA(Unported), CC BY SA(Generic) licenses are not compatible with our CC BY 4.0 license. For instance, CC BY-SA license prohibits commercial use while ours does not.So we will not be able to publish the images under CC BY-SA license. Please replace the images. Alternatively, you can seek permission from the copyright holder to publish the images under CC BY 4.0 License

See these open source resources you may use to replace images / clip-art:

- https://commons.wikimedia.org

- https://openclipart.org/"

We are thankful for the editor’s comment and suggestion. We have replaced all the images in Fig 4A and Fig 6H. These images were created by the first author Guimin Zhao himself using Adobe Illustrator 2020 software. Please refer to lines 489 and 540 of the manuscript for details.

Editor Comments:

Thank you for submitting your paper, "TRIM26 alleviates fatal immunopathology by regulating inflammatory neutrophil infiltration during Candida infection", to PLOS Pathogens. We have checked over your submission and, before we can assign your manuscript to the Editor, we ask that you first please make the following modifications:

We are thankful for the editor’s comments, and we performed the modifications suggested by the editor’ comments and concerns.

1) We notice that your supplementary figures are uploaded with the file type 'Figure'. Please amend the file type to 'Supporting Information'. Please ensure that each Supporting Information file has a legend listed in the manuscript after the references list.

We are thankful for the editor’s suggestion, and we have changed the item type of the supplementary figures to supplementary information when uploading.

2) Please ensure that the CRediT author contributions listed for every co-author are completed accurately and in full. At this stage, "All Authors" require contributions. Please ensure that the full contributions of each author are acknowledged in the ""Add/Edit/Remove Authors"" section of our submission form.

The list of CRediT author contributions may be found here:https://journals.plos.org/plospathogens/s/authorship#loc-author-contributions

We are thankful for the editor’s comment and suggestion. We have confirmed the contributions of all authors, and added the author contributions section to the manuscript of the article. Please refer to lines 644-661 of the manuscript for details.

3) We have noticed that your Financial Disclosure is duplicated in the submission.

Financial Disclosure 1: "We thank Translational Medicine Core Facility of Shandong University for consultation and instrument availability that supported this work. This work was supported by grants from the National Natural Science Foundation of China (81930039, 32230033), grants from the National key research and development program (2021YFC2300603) and the grants from Natural Science Foundation of Shandong Province (ZR201911140289, ZR2020MH165). "

Financial Disclosure 2: "The author(s) received no specific funding for this work."

Could you please confirm which of these two statements is appropriate for this submission?

We are thankful for the editor’s comment and suggestion. It was our negligence. We have made modifications to the Financial Disclosure in the Questionnaire system, which will be consistent with the manuscript. The funders had no role in study design, data collection and analysis, decision to publish, or preparation of the manuscript.

4) Thank you for stating "The data that support the findings of this study are available from the corresponding author upon reasonable request".

In the online submission form you indicate that your data is available from a co-author on this manuscript. According to our Data Policy, data must either be included with the submission, be placed in a public repository, or available from a third party contact point who must not be an author on the manuscript. Please revise your data statement to a non-author institutional point of contact, such as a data access or ethics committee, and send this to us via return email. Please also include contact information for the third party organization (this contact should be a central address and not a named person), and please include the full citation of where the data can be found.

We are thankful for the editor’s comment and suggestion. We have revised the data statement; please refer to lines 629-630 of the manuscript for details. We have uploaded Data S1. Excel spreadsheet containing, in separate sheets, the underlying numerical data and statistical analysis for Figure panels 1A, 1B, 1C, 1D, 1E, 1F, 2A, 2B, 2C, 2E, 2F, 3B, 3C, 3D, 3E, 3F, 3G, 3H, 3I, 3J, 3K, 3L, 3M, 4C, 4E, 4F, 4G, 4H, 4I, 4J, 4K, 5C, 5D, 5E, 5F, S1A, S1B, S1C, S1D, S1E, S1F, S1G, S1H, S2A, S2C, S2D, S3A, S3B, S3C, S3D, S3E, S3F, S4B, S4C, S5A, S5B, S5C, S5D, S5E, and S5F. Please refer to lines 544-549 of the manuscript for details.

5) Please upload a copy of Figure [6] which you refer to in your text on the manuscript file. Or, if the figure is no longer to be included as part of the submission please remove all reference to it within the text.

We are thankful for the editor’s comment and suggestion. It was our negligence. We have uploaded Figure 6.

6) Some material included in your submission may be copyrighted. According to PLOS’s copyright policy, authors who use figures or other material (e.g., graphics, clipart, maps) from another author or copyright holder must demonstrate or obtain permission to publish this material under the Creative Commons Attribution 4.0 International (CC BY 4.0) License used by PLOS journals. Please closely review the details of PLOS’s copyright requirements here: PLOS Licenses and Copyright. If you need to request permissions from a copyright holder, you may use PLOS's Copyright Content Permission form.

Please respond directly to this email and provide any known details concerning your material's license terms and permissions required for reuse, even if you have not yet obtained copyright permissions or are unsure of your material's copyright compatibility. Once you have responded and addressed all other outstanding technical requirements, you may resubmit your manuscript within Editorial Manager.

Potential Copyright Issues:

[Figure 4,6]: Thank you for stating" We have replaced the images in Figure 4A and Figure 6H with open source resources, all of which are from https://commons.wikimedia.org and https://openclipart.org/).

Please provide a DIRECT LINK to the source of the images or icons and their license / terms of use; [https://commons.wikimedia.org: License details]

We are thankful for the editor’s comment and suggestion. We have replaced all the images in Fig 4A and Fig 6H. These images were created by the first author Guimin Zhao himself using Adobe Illustrator 2020 software. Please refer to lines 489 and 540 of the manuscript for details.

Editor Comments:

Thank you very much for submitting your manuscript "TRIM26 alleviates fatal immunopathology by regulating inflammatory neutrophils infiltration during *Candida* infections" for consideration at PLOS Pathogens. As with all papers reviewed by the journal, your manuscript was reviewed by members of the editorial board and by several independent reviewers. The reviewers appreciated the attention to an important topic. Based on the reviews, we are likely to accept this manuscript for publication, providing that you modify the manuscript according to the review recommendations.

We are thankful for the editor’s comments, and we performed the modifications suggested by the reviewers to address the reviewers’ concerns.

1. There is not a consensus on the quality of the paper. One reviewer considers that the authors have addressed their prior concerns. A second reviewer strongly believes that while the new data do add to the quality of the paper, there are still gaps that need to be considered and addressed. It is felt and I agree that there needs to be re-writing of the manuscript to make it flow better.

We are thankful for the editor’s summarization, and we rewrote the manuscript according to logical relationship of data results and the manuscript was also revised by experts whose native language is English. The modifications were highlighted in the revised manuscript.

2. In addition, the authors need to add a paragraph regarding the limitations of their study. This will help focus the reader on what is missing and needed for future work.

We are thankful for the editor’s suggestion, and we added a paragraph regarding the limitations of our study and future work needed to be explored, and please find the content in the line 415 to 421 of the revised manuscript.

Reviewer's Responses to Questions

Part I - Summary

Please use this section to discuss strengths/weaknesses of study, novelty/significance, general execution and scholarship.

Reviewer #1: The authors have added new data supporting their findings, and responded to all my queries.

We are thankful for the reviewer’s comment.

Reviewer #3: The authors have performed additional experiments in response to the reviewers' comments. Some of the prior issues have been clarified, but some have remained unclear in the opinion of this reviewer.

We are thankful for the reviewer’s suggestion. Indeed, there are still many unresolved issues in our study, and we added a paragraph regarding the limitations of our study and future work needed to be explored, and please find the content in the line 415 to 421 of the revised manuscript.

________________________________________

Part II – Major Issues: Key Experiments Required for Acceptance

Please use this section to detail the key new experiments or modifications of existing experiments that should be absolutely required to validate study conclusions.

Generally, there should be no more than 3 such required experiments or major modifications for a "Major Revision" recommendation. If more than 3 experiments are necessary to validate the study conclusions, then you are encouraged to recommend "Reject".

Reviewer #1: (No Response)

Reviewer #3: (No Response)

________________________________________

Part III – Minor Issues: Editorial and Data Presentation Modifications

Please use this section for editorial suggestions as well as relatively minor modifications of existing data that would enhance clarity.

Reviewer #1: (No Response)

Reviewer #3: (No Response)

________________________________________

PLOS authors have the option to publish the peer review history of their article (what does this mean?). If published, this will include your full peer review and any attached files.

If you choose “no”, your identity will remain anonymous but your review may still be made public.

Do you want your identity to be public for this peer review? For information about this choice, including consent withdrawal, please see our Privacy Policy.

Reviewer #1: No

Reviewer #3: No

Overall, we are really grateful for both of the reviewers and editor’s comments and suggestions for this manuscript, which helped us to improve the quality of this study.
